# Encouraging a move toward precision geromedicine

**DOI:** 10.70401/geromedicine.2026.0020

**Published:** 2026-04-23

**Authors:** Luigi Ferrucci, Stefano Donega, Andrea B. Maier, Guido Kroemer

**Affiliations:** 1 National Institute on Aging (NIA), Intramural Research Program (IRP), National Institutes of Health (NIH), Baltimore, MD 21224, USA.; 2 NUS Academy for Healthy Longevity, Yong Loo Lin School of Medicine, National University of Singapore, Singapore 117597, Singapore.; 3 Healthy Longevity Translational Research Program, Yong Loo Lin School of Medicine, National University of Singapore, Singapore 117456, Singapore.; 4 Department of Human Movement Sciences, Faculty of Behavioural and Movement Sciences, Vrije Universiteit Amsterdam, Amsterdam Movement Sciences, Amsterdam 1081 BT, the Netherlands.; 5 Université Paris Cité, Sorbonne Université, Inserm U1138, Centre de Recherche des Cordeliers, Equipe labellisée par la Ligue contre le cancer, Paris 75006, France.; 6 Université Paris-Saclay, INSERM US23/CNRS UAR 3655, Metabolomics and Cell Biology Platforms, UMS AMMICa, Institut Gustave Roussy, Villejuif 94805, France.; 7 Institut du Cancer Paris CARPEM, Department of Biology, Hôpital Européen Georges Pompidou, AP-HP, Paris 75015, France.

**Keywords:** Geroscience, geromedicine, aging, resilience, biomarkers, trajectories, multimorbidity, healthspan

## Abstract

Aging is a heterogeneous, multi-system process driven by the interplay between accumulating molecular damage and the progressive erosion of resilience. While damage accumulates in a ubiquitous and homogeneous fashion, resilience is finite and unequally distributed across physiological systems and individuals, yielding distinct biological trajectories that diverge early in life, giving rise to the individual-specific decline in physiological function and the manifestation of a diverse spectrum of organ-specific diseases, and converge only when multisystem dysregulation overwhelms compensatory capacity. Early deviations in mitochondrial function, proteostasis, inflammation, and metabolic regulation often remain clinically silent, detectable only through gerodiagnostics, longitudinal, sensitive biomarkers of aging. Yet most biomarkers were developed to detect disease rather than quantify aging biology, and their mechanistic specificity declines with advancing multimorbidity. Precision geromedicine therefore requires the integration of gerodiagnostics that capture system-level resilience and stress responsiveness with measures of functional reserve, behavior, physiology, and the exposome, enabling the identification of individualized aging trajectories and the biological pathways that drive them. This combined approach clarifies causal pathways, enables earlier detection of vulnerability and supports individualized gerointerventions that modify aging trajectories rather than specifically but narrowly focusing on individual age-related diseases.

## 1. Introduction

Aging is constrained by the second law of thermodynamics and is therefore unavoidable. Yet the way it unfolds across organ systems and different individuals is extremely heterogeneous. Organisms can live for a long time because biology actively resists the accumulation of damage caused by entropy through maintenance, repair, compensation, and adaptive resilience. Damage accumulates continuously through metabolism, environmental exposures, and stochastic events, yet disease and functional decline do not emerge until these resilience systems begin to fail^[1–4]^. From this perspective, aging can be conceptualized as a dynamic equilibrium between the buildup of biological damage and the capacity of resilience. Like aging itself, damage is universal and unavoidable; resilience, by contrast, is finite, genetically influenced, and unevenly distributed across individuals and physiological systems. The variation of resilience across tissues and individuals generates structured heterogeneity in aging trajectories^[5,6]^. Aging, therefore, is not merely the passive accumulation of damage, but the progressive loss of balance between damaging processes and the biological mechanisms that detect, repair, and compensate for them.

As we will discuss below, this framework naturally accounts for the marked heterogeneity of aging. Damage arises largely from stochastic processes^[2]^, whereas the biological systems that counteract it (e.g., repair pathways, maintenance networks, and compensatory responses) are damage-specific and inherently incapable of addressing the full spectrum of insults^[7,8]^. Together, these features generate a wide and still insufficiently characterized spectrum of aging trajectories across different individuals^[5]^. Such heterogeneity is not fully random because physiological systems differ in their baseline operating margins. For example, the heart is at high risk of failing because it operates continuously close to critical energetic thresholds, extracting a large fraction of delivered oxygen and leaving little reserve to tolerate even modest declines in perfusion or oxygen availability^[9]^. This example illustrates how system-specific energetic constraints shape vulnerability, aligning with evidence that cardiovascular diseases are the main causes of death and, more in general, that tissues with high energetic turnover are disproportionately vulnerable to early deficits in energetic metabolism and stress-response pathways^[10]^. In addition, biological sex and gender-related exposures contribute substantially to heterogeneity in aging trajectories by shaping resilience mechanisms, biomarker profiles, and the timing and nature of system-specific decline.

From the considerations above, it emerges that biological aging is not a single linear process but the emergent result of interacting molecular, cellular, and systemic mechanisms that accumulate and compensate over time. Thus, although global aging pressure acts on all systems simultaneously, clinical phenotypes emerge preferentially in the specific physiological systems where resilience collapses first. As a result, a shared underlying biological aging process can manifest as multiple, distinct clinical expressions, depending on which systems exhaust their resilience earliest. Foundational work has shown that damage accrues continuously, while resilience mechanisms attempt to buffer its effects^[4,11]^. Recent analyses of autophagy and cellular stress-response networks further support this view, demonstrating that early declines in autophagic flux and mitochondrial turnover can shift entire physiological systems onto distinct aging trajectories long before clinical dysfunction appears^[12,13]^. In particular, human data on mitophagy and mitochondrial quality control are emerging and increasingly complement mechanistic findings from model organisms^[14]^. This framework provides the conceptual basis for understanding aging as a dynamic imbalance rather than a unidirectional decline.

## 2. The Three Operational Pillars of Geromedicine

Precision Geromedicine can be defined as an integrated framework that combines gerodiagnostics, trajectory modeling, and gerotherapeutics to identify, characterize, and modify individualized aging trajectories. Central to this concept is the recognition that aging does not proceed uniformly, but rather along distinct biological paths (“gerotypes”) that emerge from the interaction between genetic predisposition, environmental exposures, behavior, and stochastic processes over time^[15]^. These trajectories are reflected in changes across molecular, cellular, and physiological systems that can be captured through longitudinal, system-specific biomarkers of aging. For example, individuals with elevated plasma tau or related neurodegenerative markers do not transition abruptly to clinical dementia, but instead follow a progressive biological trajectory characterized by parallel changes in proteostasis, neuroinflammation, and metabolic regulation^[16,17]^. This trajectory represents a specific subset of aging biology, detectable years before clinical expression, and potentially amenable to targeted intervention. Analogously, other individuals may follow trajectories dominated by metabolic dysfunction, chronic inflammation, or impaired muscle energetics, leading to conditions such as sarcopenia or accelerated multimorbidity accumulation.

Within this framework, gerodiagnostics aims to detect and quantify these trajectories at early, preclinical stages; trajectory modeling seeks to describe their temporal evolution and identify critical inflection points; and gerotherapeutics targets the specific biological mechanisms driving each trajectory. Importantly, this approach implies that interventions are unlikely to be uniformly effective across individuals, as responsiveness depends on the underlying biology of the trajectory. Thus, Precision Geromedicine shifts the focus from disease prevention per se to the identification and modification of distinct pathways of biological aging, with the ultimate goal of preserving functional resilience and extending healthspan.

Of note, gerodiagnostics can be broadly classified into three complementary domains: molecular biomarkers of aging (e.g., epigenetic, proteomic, and metabolomic profiles), physiological, often referred to clinical, biomarkers of aging that probe system response to perturbation, and digital biomarkers of aging^[18]^ that capture functional performance and physiology in real-world settings. Among molecular tools, biological age clocks such as DNA methylation clocks, have attracted considerable attention because of their strong associations with mortality and multimorbidity. However, these measures primarily reflect accumulated biological history rather than specific causal mechanisms, and their longitudinal dynamics, mechanistic interpretation, and incremental predictive value beyond standard clinical assessments remain incompletely defined. At present, their role is therefore better viewed as exploratory rather than directly actionable in clinical settings. This Perspective aims to outline the conceptual and methodological advances required to transform such theoretical tools into clinically meaningful components of precision geromedicine.

## 3. Why Heterogeneity Makes Precision Non-Negotiable

The concept that aging does not manifest uniformly across individuals is a central challenge in geroscience. Longitudinal cohort studies, systems-level analyses, and multi-omics profiling consistently show that genetics, early-life stressor exposures, metabolic states, and chronic inflammatory burden shape distinct biological trajectories in the early life stages of different individuals^[6,15]^. Physiological studies of metabolic flexibility and muscle energetics further demonstrate that these early divergences are detectable at the level of system-specific performance, with measurable differences in mitochondrial efficiency and substrate utilization emerging decades before the manifestation of the disease^[19]^. These trajectories influence which systems fail first, how rapidly resilience erodes, and which diseases emerge decades later. Therefore, recognizing this structured heterogeneity is essential for developing a precision framework for aging biology, as previously published^[20]^: we extend this framework by emphasizing the dynamic balance between damage and resilience, the time-dependent interpretation of biomarkers, and the divergence–convergence structure of aging trajectories.

Particularly important, we pose that resilience operates across multiple biological scales and should be distinguished accordingly. At the biological level, resilience encompasses molecular and cellular mechanisms that prevent, repair, or compensate for damage, including DNA repair, proteostasis, and mitochondrial quality control. At the phenotypic level, resilience reflects system-level buffering capacity, whereby redundancy and network interactions limit the functional impact of localized deficits. At the functional level, resilience refers to the organism’s ability to maintain performance and autonomy through physiological or behavioral adaptation despite underlying impairments^[21]^. Several lines of research suggest that resilience can be operationalized independently of clinical outcomes as a property of system dynamics. Measurable features include recovery kinetics following perturbation, intra-individual variability over time, and the slope and magnitude of responses to standardized stressors. These metrics can be applied across biological scales, enabling prospective quantification and comparison of resilience across individuals and systems.

This new knowledge has pushed the research on aging biology toward a conceptual transition. Once treated as a background condition characterized by an inevitable process that merely increases disease risk, aging is now increasingly understood as a measurable, causal, and potentially modifiable biological process. This paradigm shift carries profound implications for medicine. If aging biology lies upstream of most age-related diseases and functional decline, then intervening on aging itself becomes not only plausible but necessary. Yet this recognition immediately exposes a central tension: while aging is universal, its clinical and biological manifestations are strikingly heterogeneous.

In fact, individuals manifest age-related diseases in different ways. Some develop osteoarthritis early and remain cognitively intact into advanced age; others experience metabolic dysfunction or neurodegeneration decades before musculoskeletal failure. Even when viewed purely through clinical manifestations, aging does not follow a single, uniform trajectory but instead unfolds along multiple paths shaped by early-life factors, lifestyle, and chronic conditions. For example, individuals with obesity or diabetes experience age-related metabolic changes and health outcomes that differ substantially from those of lean, metabolically healthy individuals^[22]^. Similar distinctions likely apply to conditions characterized by prolonged exposure to environmental stressors^[23]^ and by chronic inflammation/infection (e.g., due to human immunodeficiency virus) producing persistent immune activation and an ‘inflammaging’ phenotype with distinct biomarker patterns^[24,25]^. While these trajectories are often defined by observable phenotypes, others may be rooted at deeper biochemical, metabolic, or energetic levels, long before overt clinical disease becomes apparent.

These divergent aging trajectories provide support in explaining why age-related diseases vary widely in their timing of onset, sequence of appearance, and organ involvement. During early and mid-adulthood, specific pathophysiological mechanisms, such as insulin resistance, ectopic fat accumulation, mitochondrial dysfunction, or chronic immune activation, tend to dominate risk, producing highly heterogeneous patterns of morbidity across individuals. Evidence from mobility-related science research further supports this view: longitudinal assessments of muscle energetics and physical performance reveal stable, individualized trajectories that predict frailty and functional decline, underscoring that physiological divergence is not merely biochemical but also functional^[26,27]^.

## 4. Genetic and Early-Life Drivers of Divergent Trajectories

Part of the divergence of *gerogenesis,* the lifelong process through which biological aging unfolds across interacting molecular, cellular, and physiological systems, into distinct aging trajectories arises from life-compatible genetic variation summarized as gerogenes (i.e., genes that favor the aging process) and facilitated by the failure of gerosuppressive genes and processes that counteract aging^[28]^. Genetic diseases are typically viewed as binary (e.g., present or absent), but this framework applies primarily to mutations that cause overt dysfunction and reduced survival. On the other hand, mild or partial genetic variants may activate adaptive responses that alter biological trajectories across the life course. For example, rare heterozygous variants in insulin-like growth factor 1 receptor *(IGF1R)* are enriched among centenarians^[29]^. Individuals carrying these variants tend to be smaller and display altered metabolic profiles, a phenotype that mirrors the healthspan benefits seen in other human defects of the Growth Hormone/insulin-like growth factor 1 *(IGF-1)* axis^[30]^. These trajectories are accompanied by increased forkhead box O *(FOXO)* activity and improved stress resistance^[31]^, as well as improved proteostasis^[32]^. Of note, these effects are not limited to late life, since growth, metabolism, and stress-response systems are remodeled from early development onward, ultimately manifesting as delayed aging.

Similar principles apply to cardiometabolic aging. Partial loss-of-function variants in apolipoprotein C-III (APOC3) and angiopoietin-like protein 3 (ANGPTL3) associate with lower triglycerides and reduced cardiometabolic risk^[33]^, whereas proprotein convertase subtilisin/kexin type 9 (PCSK9) loss-of-function variants (for example R46L) reduce low-density lipoprotein (LDL) cholesterol and are associated with lower cardiovascular risk^[34,35]^. These variants induce persistent shifts in lipid handling, insulin sensitivity, and inflammatory status, engaging pathways such as 5’ AMP-activated protein kinase *(AMPK*) activation, autophagy, and reduced inflammasome signaling. Consequently, the aging trajectory of cardiometabolic systems is altered decades before clinical manifestations of aging appear evident. Additional examples illustrate this principle: lifelong alterations in lipid handling from cholesteryl ester transfer protein *(CETP)* loss-of-function variants^[36]^; reduced inflammation and improved insulin sensitivity in carriers of interleukin-6 receptor (*IL6R*) Asp358Ala^[37]^; lower lifetime cardiovascular risk in carriers of lipoprotein(a) gene (LPA) variants that reduce lipoprotein(a) levels^[38]^; and heterogeneous associations between endothelial nitric oxide synthase (eNOS) Glu298Asp and vascular outcomes^[39]^.

Altogether, these variants reshape metabolic, inflammatory, and vascular pathways from early adulthood onward, producing distinct cardiometabolic aging trajectories. In addition to these molecular examples, physiological studies demonstrate that genetically influenced differences in mitochondrial efficiency and muscle energetics can be detected decades before overt disease, reinforcing that genetic variation shapes not only biochemical pathways but also system-level performance^[40]^.

The aforementioned examples likely only represent the tip of a yet-to-be discovered iceberg of polymorphisms affecting the development of age-related diseases. It can be suspected that small-effect genetic variants, particularly those affecting systems that surveil, repair, and respond to molecular damage, can generate distinct trajectories of biological aging. These trajectories are further shaped by environmental and behavioral exposures as found for tobacco exposure, accelerating epigenetic aging signatures in multiple cohorts; epigenome-wide studies show dose-dependent methylation changes and epigenetic age acceleration that partially reverse after smoking cessation^[41,42]^. The characterization of these trajectories has important therapeutic implications. Interventions that are effective in one biological aging context may fail in another. Thus, identifying, characterizing, and linking distinct aging trajectories to pathology represents both the greatest challenge and the greatest opportunity for precision geromedicine^[20]^. For example, pharmacological approaches and regenerative strategies are being explored to restore tissue integrity, including biomaterial-based approaches that modulate the local microenvironment and support repair processes, as well as interventions targeting the interaction between inflammation and angiogenic signaling in degenerative conditions^[43–45]^.

As aging progresses, however, the underlying biological mechanisms increasingly converge toward a shared state characterized by declining physiological reserve, multisystem dysregulation, and heightened vulnerability to stressors. This convergence forms the biological substrate of frailty^[46]^. Although frailty may arise from diverse initiating causes, its downstream biology appears remarkably stereotyped. Consistent with this view, it was proposed that once the frailty syndrome is established, its progression often shows convergent features characterized by multisystem dysregulation and declining physiological reserve, although partial and context-dependent reversibility has been reported^[47–49]^.

The divergence–convergence framework generates several testable predictions. First, inter-individual variability in system-specific biomarkers and physiological function should increase during early and mid-life, reflecting divergence, and subsequently decrease in late life as multisystem dysregulation accumulates and trajectories converge. Second, within-individual trajectories should exhibit relative stability over time, with system-specific slopes that precede and predict clinical outcomes. Third, as convergence progresses, biomarkers are expected to lose mechanistic specificity and increasingly reflect generalized dysregulation rather than pathway-specific processes.

Indirect evidence supporting convergence emerges across multiple levels. At the molecular level, dysfunction in specific resilience mechanisms (e.g., impaired mitochondrial function) can propagate across interconnected networks, leading to secondary failures in proteostasis, cellular senescence, extracellular matrix remodeling, and inflammation, consistent with a progressive loss of stress-response signaling across organs^[14,50]^. At the phenotypic level, epidemiological studies show that after the end of health expectancy, the accumulation of multimorbidity follows approximately linear trajectories with limited disease specificity, suggesting a shift from pathway-specific processes to generalized vulnerability^[51]^. At the functional level, once compensatory strategies fail, physiological systems lose the ability to maintain performance through adaptation, and decline reflects a more global loss of homeostatic regulation^[5]^, which is also consistent with the geriatric imperative that health in older persons is best assessed in functional terms^[52]^.

These predictions will be best evaluated using longitudinal data with repeated within-person measurements, allowing separation of true biological trajectories from cohort or measurement effects. The framework would be challenged by evidence of persistent or increasing heterogeneity in advanced age, or by the absence of stable, system-specific trajectories earlier in life.

Finally, the apparent paradox of a single aging process driving manifold disease has often been interpreted as an artifact of measurement or biological complexity. We argue instead that heterogeneity is not incidental but the defining feature of heterogenous human aging biology. Recognizing this is essential for the development of gerodiagnostics capturing the differences in aging phenotypes and trajectories and for the development of effective gerointerventions. Accordingly, precision in Geromedicine is not an aspirational refinement. It is instead a biological requirement.

## 5. A Time Course Perspective

Longitudinal studies reveal that differences in metabolic flexibility, inflammatory tone, mitochondrial efficiency, and DNA repair capacity drive distinct biological trajectories^[6,53]^. These trajectories remain stable for decades before converging later in life as multisystem dysregulation accumulates. This life-course perspective clarifies why early-life and mid-life biology exert disproportionate influence on late-life outcomes. Early in life, resilience mechanisms buffer damage across tissues, preserving functional reserve, and masking underlying biological strain. Over time, genetically encoded differences in repair, adaptation, and compensation (further shaped by cumulative environmental and behavioral exposures) lead to divergence in aging trajectories. Such convergence reflects shared burden, not shared mechanism: the biological systems that fail first, and the timing of their failure, remain distinct. This temporal structure reinforces the idea that aging is not only “faster” or “slower” across individuals but instead unfolds along qualitatively different paths shaped by early-life biology, mid-life exposures, and system-specific resilience thresholds. Aging trajectories can be conceptualized as longitudinal paths describing the evolution of biological systems over time, rather than as static deviations from a norm. In this framework, each trajectory is defined by measurable features, including its baseline state, rate of change (slope), variability, and the presence of inflection points reflecting shifts in underlying biology. Different physiological systems within the same individual may follow partially independent trajectories, which interact and may compensate for one another until compensatory capacity is exhausted.

Importantly, trajectories are not observable from cross-sectional data but must be inferred from repeated longitudinal measurements using approaches such as mixed-effects modeling, clustering of individual slopes, or latent class trajectory analysis. This formalization allows trajectories to be treated as quantifiable biological entities that can be compared across individuals, linked to outcomes, and potentially modified by intervention. We recognize that some of the evidence discussed here derives from cross-sectional analyses, which are valuable for identifying associations and generating hypotheses. We anticipate that biomarkers that show weak or inconsistent associations with outcomes in cross-sectional analyses may demonstrate strong predictive value when evaluated longitudinally through within-individual changes or variability over time. For instance, cross-sectional snapshots of individual circulating biomarkers often exhibit high inter-individual noise and confounding from acute stressors. However, combining these markers to quantify systemic physiological dysregulation (specifically evaluating the individual’s longitudinal rate of change over time) reveals an accelerating trajectory that serves as a highly robust predictor of incident frailty and mortality^[54,55]^. This is consistent with the notion that tracking changes in biomarkers reflects the underlying mechanisms of biological aging.

**Figure 1.**
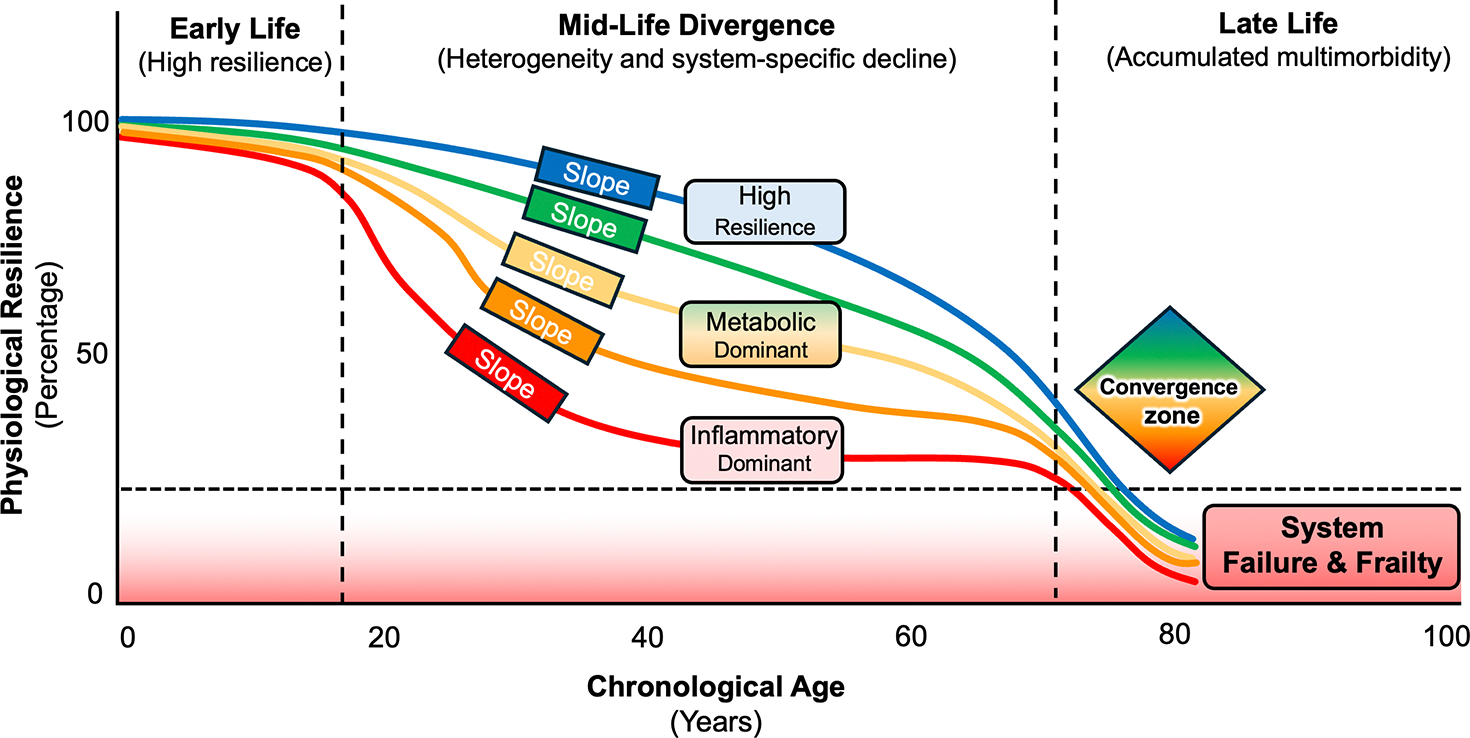
Lifespan trajectories of physiological resilience. Early life shows uniformly high resilience across individuals. By mid-life, trajectories diverge as system-specific vulnerabilities emerge, producing distinct decline slopes (e.g., high-resilience, metabolic-dominant, and inflammatory-dominant pathways). These variable mid-life slopes reflect differences in genetic load, early-life exposures, and organ-system thresholds. In late-life, accumulated multisystem damage drives all trajectories toward a shared convergence zone, where resilience collapses below the frailty threshold and multimorbidity becomes universal. The structure highlights mid-life as the critical intervention window, when modifying pathway-specific decline rates may still alter long-term outcomes.

The lifespan trajectories of aging expressed as “divergence and convergence” of physiological resilience are represented in Figure 1. Physiological studies of movement and energetics support this temporal framework: longitudinal measures of muscle power, gait energetics, and metabolic flexibility show individualized, predictable slopes that anticipate later frailty and functional decline^[19]^. To move beyond association and identify when interventions are most likely to alter aging trajectories, studies should combine repeated longitudinal measurements with causal-inference tools and experimental validation^[56]^. Among these strategies, we mention mixed-effects models to estimate within-person slopes, Mendelian randomization to probe upstream mechanisms when valid instrumental variables exist, longitudinal mediation to map temporal ordering of intermediate biomarkers, and randomized proof-of-concept trials that include mechanistic endpoints^[57]^. Stimulus–response physiological tests (e.g., mitochondrial recovery after exercise, vascular reactivity, or recovery from a glucose-challenge) operationalize resilience and temporal measurement concepts^[58]^. Together, these approaches help distinguish upstream aging biology from downstream disease effects and identify the time windows when modifying a pathway is plausibly causal.

Therefore, in aging, time is not merely a covariate but a structural dimension that is best understood as progression along finite, partially convergent trajectories, rather than as a single process unfolding at different speeds^[21]^.

## 6. The Silent Phase of Aging Biology

A defining feature of aging biology is that the earliest deviations from homeostasis occur long before clinical disease becomes apparent. As a fact, much of aging biology unfolds silently. These “silent” phases involve subtle mitochondrial inefficiency, impaired proteostasis, low-grade inflammation, and shifts in metabolic signaling that can be detected only through sensitive biomarkers or multi-omics profiling™. During this period, compensatory mechanisms remain active, masking functional decline despite accumulating molecular stress. Evidence from longitudinal proteomic and metabolomic studies shows that coordinated shifts in inflammatory mediators, mitochondrial metabolites, and proteostasis-related proteins appear years before clinical disease^[59,60]^. Although metabolomic measurements are highly sensitive to pre-analytical conditions, necessitating rigorous standardization, early mitochondrial dysfunction can nevertheless be detected through circulating acylcarnitines^[61]^ and age-associated alterations in tricarboxylic acid cycle (TCA-cycle) intermediates observed in aging cohorts and targeted metabolomics studies^[62]^. In parallel, senescent cell burden^[63]^ and components of the senescence-associated secretory phenotype rise decades before the onset of frailty^[64]^.

Crucially, the interpretation of these biomarkers is time-dependent. Early in life, they primarily capture upstream biological mechanisms, such as proteostasis, mitochondrial function, inflammation, or cellular senescence, and thus retain mechanistic clarity that may be captured by circulating biomarkers. Later in life, as clinical disease emerges, these same markers are increasingly shaped by downstream pathology, its systemic effects, and gerointervention. Their prognostic strength may persist, but their capacity to inform underlying mechanisms progressively erodes. This is consistent with the clinical notion that older people’s health is better characterized by functional measures rather than by biomarkers or disease diagnoses^[21]^. Biological clinical, and digital^[18]^ biomarkers of aging^[65]^ should not be seen as competitors but rather as complementary, yielding optimal metrics in different situations. This distinction helps explain why many biological clocks perform well prognostically but offer limited insight into causality or intervention targets^[66]^. This pattern is well documented in studies showing that epigenetic clocks predict mortality and weakly predict frailty^[67]^ but associate strongly with comorbidity burden in late life^[68,69]^, and that inflammatory biomarkers lose pathway specificity as multimorbidity accumulates^[70]^.

Similarly, circulating markers of oxidative stress such as F2-isoprostanes^[71]^, protein carbonyls, and oxidized lipids^[72]^ associate strongly with mitochondrial reactive oxygen species (ROS) production and antioxidant capacity in mid-life, but in later life they increasingly reflect the burden of comorbidities, medication exposure, and systemic inflammation. Likewise, markers of proteostasis such as circulating chaperones or ubiquitinated proteins are mechanistically interpretable earlier in life but become progressively nonspecific in advanced multimorbidity. These patterns underscore that biomarkers are not static indicators but rather dynamic readouts whose interpretation depends critically on the biological and clinical context in which they are measured (see Section 7).

The implication is straightforward, though often overlooked: in aging research and intervention, timing matters more than biomarker choice. Consequently, we should not be misled by the results of cross-sectional studies. Cross-sectional studies dominate human aging research, but they are poorly suited to capturing aging biology. By collapsing time, they exaggerate heterogeneity and obscure structure. What appears incoherent in a snapshot often resolves into patterned life-course trajectories when individuals are followed longitudinally. Longitudinal analyses from cohorts such as DunedinPACE, InCHIANTI, and the Baltimore Longitudinal Study of Aging demonstrate that individual biomarker trajectories (rather than single measurements) predict functional decline, frailty, and mortality^[73–75]^.

Longitudinal approaches reveal stable individual paths linking early biological changes to later disease onset and functional decline. They expose the temporal ordering of biological stress, compensatory failure, and clinical expression. Research on biomarkers of aging may lead to advancements that translate this science toward clinical applications. However, this shift requires more understanding of how biomarkers change over time, their stability in repeated measures in a certain time frame, and the predictive validity of such changes independent of their average value. Studies of within-person biomarker variability show that short-term fluctuations in inflammatory, metabolic, and endocrine markers predict hospitalization and mortality independent of mean levels^[76]^. These findings reinforce that temporal dynamics (not merely static values) carry the mechanistic information needed for precision geromedicine. Without this temporal dimension, precision in aging medicine cannot be achieved.

**Figure 2.**
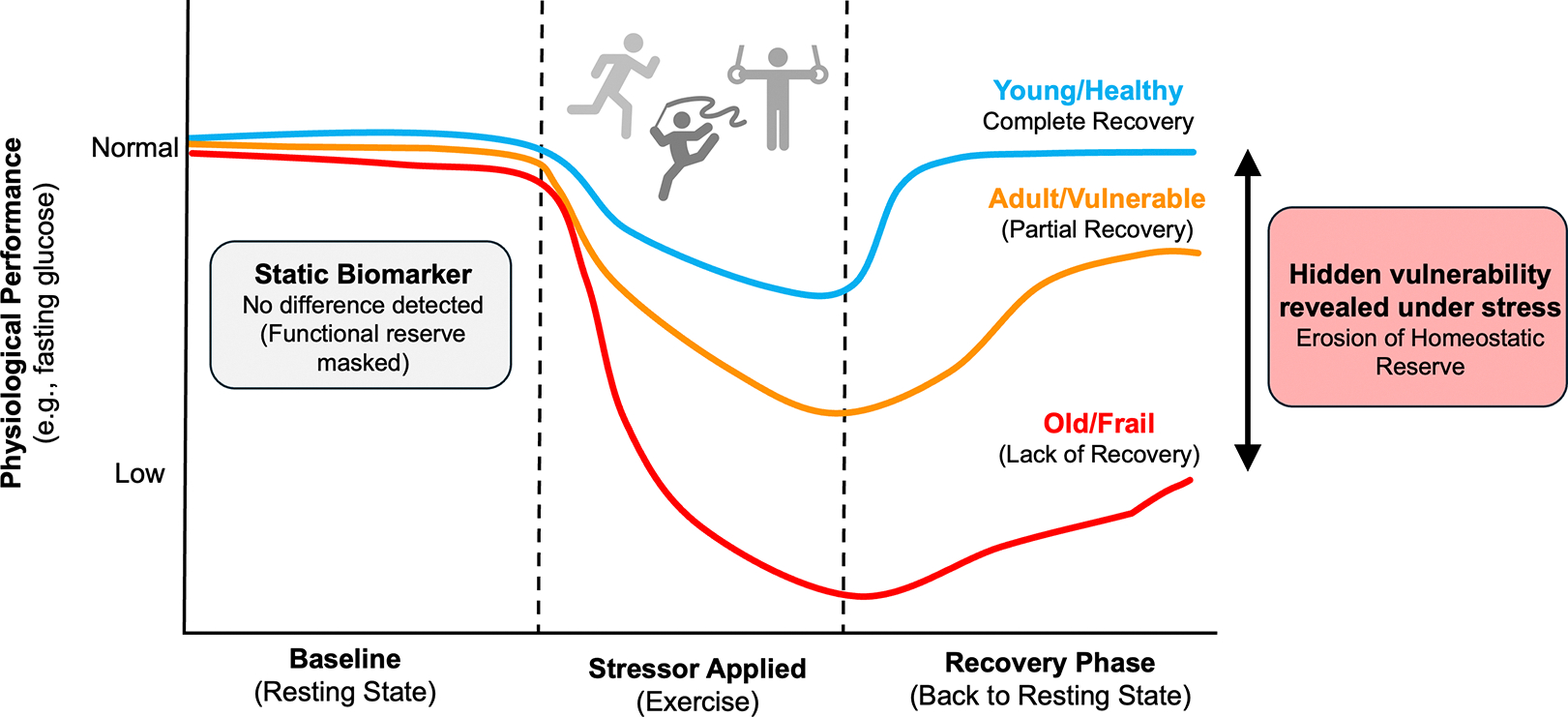
Exposing hidden vulnerability: Static biomarkers versus dynamic physiological stress tests. Static biomarkers assess physiological systems at rest, where compensatory mechanisms may mask declining reserve. In contrast, a controlled perturbation reveals differences in system resilience. A healthy individual shows rapid recovery, a vulnerable individual shows delayed or incomplete recovery, and a frail individual fails to recover. These response dynamics expose early functional deficits not detectable in basal conditions.

To advance precision geromedicine, biological biomarker-based approaches must be complemented by clinical and digital biomarkers of aging that directly quantify system-specific integrity. Measures such as vascular endothelial reactivity, autonomic responsiveness, metabolic flexibility, and muscle energetics offer functional readouts of aging processes^[40,77]^. Because physiological tests probe how systems respond to perturbation, they provide a more mechanistic window into resilience and early system-level decline^[57]^. This principle is reinforced by movement-science research showing that early declines in muscle energetics can be detected through exercise-based assessments long before clinical impairment^[69]^. Figure 2 further illustrates this hidden vulnerability by contrasting static biomarkers with a dynamic framework based on physiological stress testing.

## 7. Box 1: Exploring the Gap Between Biological Age and Functional Resilience

Current metrics of aging generally fall into two categories that provide fundamentally different types of information yet are often conflated in geroscience: measures of accumulated damage (biological history) and measures of functional resilience (future capacity). Most “aging clocks” and molecular biomarkers capture the system in a basal (resting) state. Whether based on DNA methylation patterns^[78]^, proteomic signatures^[60]^, or inflammatory cytokines^[70]^, these metrics primarily reflect the cumulative history of molecular insults and the system’s compensatory efforts to maintain homeostasis. While these static markers are excellent for predicting long-term mortality and multimorbidity risk^[15]^, they often lose mechanistic specificity in late life. In advanced age, basal biomarkers cannot easily distinguish between primary aging processes and the secondary consequences of established disease, nor can they quantify how much functional “buffer” a system has left before failure. Notably, this limitation is partially overcome by pace-of-aging measures (e.g., DunedinPACE) that differ from static epigenetic clocks in that they estimate the rate of biological change over time, rather than the accumulated biological state at a single time point.

In contrast, resilience, defined as the capacity to recover from perturbation, is frequently invisible at rest^[79]^. A physiological system operating near failure often appears normal in a basal state because compensatory mechanisms are working in overdrive; the deficit is revealed only when the system is challenged. This stimulus-response paradigm argues that the true phenotype of aging is not the state of the system at rest, but the dynamics of its recovery^[80]^. Physiological “stress tests” that quantify recovery dynamics such as fasting glucose, c-reactive protein (CRP), vascular response to shear stress (and many more not here listed) therefore provide complementary, mechanistic information that basal biomarkers cannot^[27]^. These functional readouts likely detect the erosion of homeostatic reserve long before resting biomarkers become abnormal. Precision geromedicine should therefore integrate both types of measures: molecular clocks to map accumulated history and standardized stimulus–response tests to quantify remaining functional reserve and identify the systems most amenable to early intervention.

## 8. Precision Geromedicine is a Biological Necessity

The growing recognition that aging is heterogeneous, multi-systemic, and time-structured has led to a shift from population-level interventions toward individualized strategies that target the specific biological processes failing first in each person in clinical settings^[81]^. Precision geromedicine draws on principles from oncology, immunology, and systems biology, where patient-specific molecular profiles guide therapeutic decisions^[82 83]^. Applying similar logic to aging requires identifying early deviations in resilience pathways (e.g., mitochondrial quality control, proteostasis, DNA repair, metabolic flexibility, and inflammatory regulation) and intervening before compensatory mechanisms collapse.

**Figure 3.**
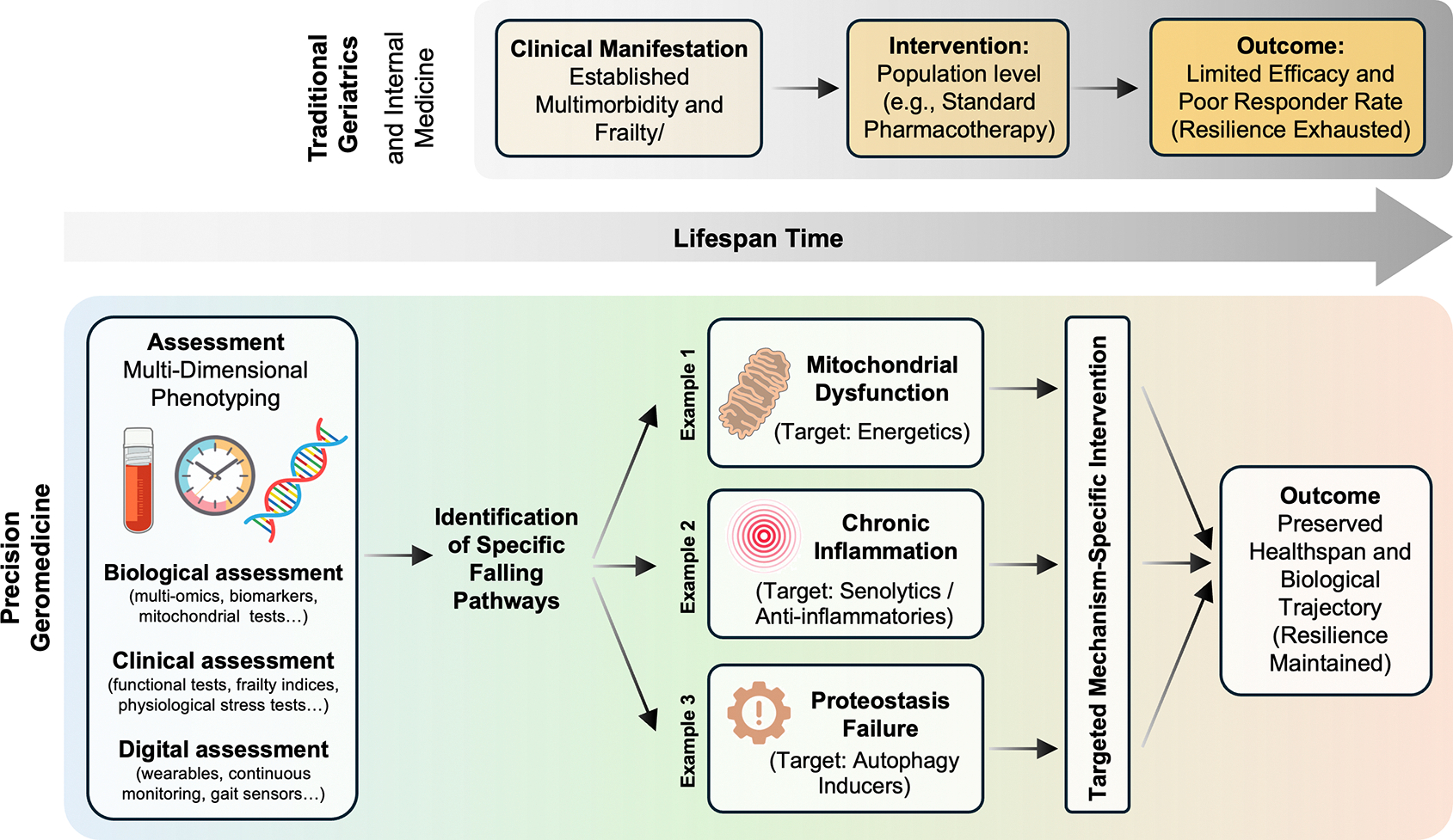
The paradigm shift to precision geromedicine: Targeting trajectories rather than endpoints. Traditional medicine (top) intervenes late, often after convergence into multimorbidity and frailty, when physiological resilience is largely exhausted. In contrast, precision geromedicine (bottom) targets earlier phases of trajectory divergence by integrating molecular, physiological, and functional data to identify dominant failing pathways (e.g., mitochondrial dysfunction, inflammation, proteostasis). This approach enables mechanism-based, individualized interventions aimed at modifying aging trajectories and preserving functional resilience.

Precision geromedicine follows naturally from these observations, as depicted in Figure 3. If aging unfolds heterogeneously, along multiple trajectories shaped by differential resilience, then interventions must be matched to the biology that is failing first and to the time at which failure remains preventable. Intervening on population averages dilutes effects, obscures mechanisms, and risks acting too late, when biology has already converged toward irreversible decline. Precision geromedicine therefore aims to identify vulnerable systems before compensatory capacity is exhausted, and to alter trajectories rather than endpoints. This is not a call for ever finer stratification for its own sake, but a recognition that biological heterogeneity is intrinsic to aging itself.

These considerations have direct implications for intervention studies in aging. Given the complexity, heterogeneity, and partial convergence of aging trajectories, it is likely that early clinical studies or trials targeting aging biology will produce modest or even negative results. Such outcomes should not be interpreted as failure of the underlying biological concept, but rather as evidence of our still-limited understanding of how and when to intervene in a complex, multi-system process.

A potential implementation pathway for precision geromedicine can be conceptualized as an iterative process rather than a fixed clinical algorithm. First, longitudinal multidimensional data (molecular, physiological, and functional) are collected to identify candidate aging trajectories. Second, individuals are stratified according to dominant biological signatures (e.g., inflammatory, metabolic, or senescence-related profiles). Third, targeted interventions are deployed based on these signatures, including candidate gerotherapeutics such as senolytics, mechanistic target of rapamycin (mTOR) modulators, metabolic interventions, or lifestyle-based approaches. Finally, heterogeneity in treatment response is analyzed to refine trajectory definitions and improve patient stratification. In practice, implementation of this framework would require stratifying individuals based on integrated, multidimensional data rather than single markers. This could include molecular profiles (e.g., inflammatory, metabolic, or senescence-related biomarkers), physiological assessments that capture system response to perturbation, and functional measures of performance. Within this context, “failing pathways” are inferred from consistent patterns across domains, for example, concordant evidence of impaired mitochondrial function, reduced metabolic flexibility, and altered recovery dynamics.

At a minimum, this approach would require repeated longitudinal measurements to estimate within-individual trajectories, complemented by cross-sectional assessments to define current system status. While such datasets are not yet widely available in clinical practice, emerging longitudinal cohorts and deeply phenotyped studies provide a foundation for this approach. We recognize that at present, this framework remains largely investigational. For example, a plausible near-term strategy would be to embed gerotherapeutic interventions within longitudinally characterized cohorts and evaluate responder versus non-responder profiles. In the case of senolytics, one might hypothesize that individuals with evidence of increased senescent cell burden or related phenotypes would derive the greatest benefit. Such studies would not only test intervention efficacy but also help define the biological features that characterize actionable aging trajectories.

Early geromedicine trials illustrate this challenge. For example, the Participatory Evaluation of Aging with Rapamycin for Longevity Study (PEARL) trial (ClinicalTrials.gov ID NCT04488601) highlights heterogeneity in baseline biology^[84]^, while low-dose target of rapamycin complex 1 (TORC1) inhibition trials demonstrate immune modulation and variable responses^[85]^. Supplementation with (nicotinamide mononucleotide; NMN) improves gait speed and quality of life^[86]^ (ClinicalTrials.gov, NCT04823260), but responses are heterogeneous, warranting NAD+ measurements to tailor personalized dosage regimes and optimize NMN utilization^[87]^. Senolytic pilot studies show context-dependent effects modulated by baseline inflammation and metabolic status^[88]^.

Parallel evidence from autophagy-targeting interventions indicates that variability in baseline mitophagy efficiency and stress-response capacity strongly influence responsiveness to interventions aimed at improving cellular quality control^[12,50]^.

Importantly, well-designed studies can remain highly informative even when primary clinical endpoints are not met. Trials in aging should therefore be constructed to capture changes across multiple physiological systems and biological pathways, extending beyond the specific mechanism hypothesized to be targeted by the intervention (see Section 9). Multidimensional phenotyping (including molecular, metabolic, inflammatory, functional, and physiological measures) can reveal unanticipated effects, compensatory responses, or context-dependent benefits that would otherwise remain invisible. Systems-level analyses in intervention trials show that pathway-level responses (such as shifts in mitochondrial respiration, immune remodeling, or metabolic flexibility) often precede or occur independently of clinical outcomes^[89,90]^. These multidimensional readouts are essential for elucidating how interventions reshape aging trajectories. Interventions such as metformin, rapamycin^[91]^, or NAD+ precursors influence multiple hallmarks of aging simultaneously (mitochondrial function, autophagy, inflammatory signaling, and metabolic regulation) yet their effects vary markedly across individuals depending on baseline metabolic state, inflammatory burden, and mitochondrial efficiency^[92,93]^. Altogether, these findings emphasize that intervention effects must be interpreted within the broader biological context of each participant’s aging trajectory.

## 9. Box 2: Standardization and Rigorous Biobanking

To enable comparability across trials and sites, physiological stress tests and mechanistic assays require harmonized protocols, central training, pre-trial calibration studies and ongoing quality control. Minimal reporting should include stimulus parameters, pre-test conditions (fasting, medications), timing of sampling, instrument calibration and analytic pipelines. Pre-trial reproducibility studies and central reading (where feasible) reduce measurement error and permit pooled analyses across cohorts^[57,77]^. Physiological heterogeneity further modulates intervention response: individuals with preserved metabolic flexibility or higher mitochondrial respiratory capacity often exhibit more robust improvements in functional outcomes, reinforcing the need to integrate physiological profiling into trial design^[94]^. Capturing these multidimensional responses is essential for understanding who benefits, who does not, and why. Precision geromedicine risks widening disparities if multi-omics profiling and physiological stress testing remain concentrated in well-resourced centers. Trials should therefore prioritize diverse recruitment, pragmatic stress-test protocols, validated low-cost surrogates and embedded implementation research to ensure equitable translation across populations and health systems^[82]^. Equally important is a rigorous and standardized biobanking: collect and document blood (EDTA, serum), urine, peripheral blood mononuclear cells (PBMCs) and, when feasible, tissue biopsies; record fasting status, time of day, medication use and other pre-analytic metadata; aliquot to minimize freeze–thaw cycles and store at −80 °C for short-term or in liquid nitrogen for long-term preservation; maintain harmonized standard operating procedures (SOPs) and a searchable metadata registry so samples and metadata are interoperable across studies and available for future reanalysis as technologies and hypotheses evolve. Well-curated biobanks transform individual trials from isolated experiments into durable resources, allowing past interventions to be reinterpreted in light of evolving models of aging biology, enabling retrospective mechanistic analyses that increase the value of both positive and negative studies and accelerate equitable translation.

## 10. Conclusion

The emerging synthesis across geroscience, systems biology, and longitudinal epidemiology points toward a unifying principle: aging is not a single process, but a structured set of trajectories shaped by damage, resilience and time. Precision geromedicine seeks to map these trajectories, identify early inflection points, and intervene before irreversible decline occurs. Accepting aging as a causal, heterogeneous, and time-structured biological process leaves little ambiguity about the path forward. A single, uniform approach to aging intervention is biologically incoherent. We recognize that significant challenges remain, including the need for standardized measurements, scalable longitudinal data collection, and integration of multidimensional datasets across diverse populations. However, we are convinced that precision is not optional: it is biologically required, and therefore mandatory.

The future of geroscience lies not in asking whether we can target aging, but in understanding which aging, in whom, and when to facilitate the transition to precision geromedicine. Only by embracing heterogeneity as signal rather than noise can aging biology be translated into effective, preventive, and truly personalized medicine.
